# Computational Tools and Resources Supporting CRISPR-Cas Experiments

**DOI:** 10.3390/cells9051288

**Published:** 2020-05-22

**Authors:** Pawel Sledzinski, Mateusz Nowaczyk, Marta Olejniczak

**Affiliations:** Department of Genome Engineering, Institute of Bioorganic Chemistry, Polish Academy of Sciences, Noskowskiego 12/14, 61-704 Poznan, Poland; psledzinski@ibch.poznan.pl (P.S.); matnowaczyk@ibch.poznan.pl (M.N.)

**Keywords:** genome editing, CRISPR, database, off-target, sgRNA, microhomology, NHEJ, HDR, predictive algorithms, sgRNA design, NGS analysis

## Abstract

The CRISPR-Cas system has become a cutting-edge technology that revolutionized genome engineering. The use of Cas9 nuclease is currently the method of choice in most tasks requiring a specific DNA modification. The rapid development in the field of CRISPR-Cas is reflected by the constantly expanding ecosystem of computational tools aimed at facilitating experimental design and result analysis. The first group of CRISPR-Cas-related tools that we review is dedicated to aid in guide RNA design by prediction of their efficiency and specificity. The second, relatively new group of tools exploits the observed biases in repair outcomes to predict the results of CRISPR-Cas edits. The third class of tools is developed to assist in the evaluation of the editing outcomes by analysis of the sequencing data. These utilities are accompanied by relevant repositories and databases. Here we present a comprehensive and updated overview of the currently available CRISPR-Cas-related tools, from the perspective of a user who needs a convenient and reliable means to facilitate genome editing experiments at every step, from the guide RNA design to analysis of editing outcomes. Moreover, we discuss the current limitations and challenges that the field must overcome for further improvement in the CRISPR-Cas endeavor.

## 1. Introduction

The CRISPR-Cas (clustered regularly interspaced short palindromic repeats-CRISPR-associated protein) system has become a cutting edge technology that has revolutionized the field of genome engineering. The use of Cas9 nuclease is currently the method of choice in most tasks requiring a specific DNA cleavage due to its low cost, relative ease of use and straightforward applications. In its natural context, the CRISPR-Cas9 system builds an anti-mobile genetic element adaptive immunity of bacteria and archaea. It stores the record of, e.g., viral infections in the prokaryotic genome in the form of CRISPR arrays composed of acquired fragments of viral DNA (called spacers) separated by palindromic repeats. Transcription of the array and subsequent processing of the transcript leads to formation of a CRISPR-associated RNA (crRNA) or, simply, guide RNA (gRNA). Its distinguishing feature is a strict complementarity of its part containing the spacer to the original fragment of the viral genome (protospacer). crRNA anneals to the separately transcribed trans-activating crRNA (tracrRNA) and then forms a ribonucleoprotein complex (RNP complex) with the Cas nuclease. The crRNA guides the complex to any complementary sequence with a directly adjacent trinucleotide protospacer-adjacent motif (PAM, NGG in case of SpCas9) in the genome of an invading element. PAM is located directly downstream of the protospacer and plays a critical role in the process of self vs. non-self discrimination preventing autoimmune response. Subsequently, the Cas9 nuclease introduces a double-strand break (DSB) 3 bp upstream of the PAM, leading to inactivation of an invader [1].

To simplify the system in the laboratory setting, a chimeric RNA molecule containing crRNA and tracrRNA, which is transcribed as a single guide RNA (sgRNA), is commonly applied [2]. Therefore, the basic version of the system requires only two components, namely, sgRNA and Cas9 nuclease, which both can be expressed from a single plasmid in cells to be edited. Alteration of the nucleotide composition of the gRNA allows the system to target virtually any sequence in the genome and specifically introduce a DSB [1]. The break is subsequently repaired either by nonhomologous end joining (NHEJ) or by homology-directed repair (HDR). The NHEJ pathway results in small insertions or deletions (indels) traditionally considered random, but recent evidence suggests that the outcomes are predictable to a certain extent [3,4,5,6,7]. This pathway is frequently utilized to induce frameshift mutations that lead, consequently, to the knockout (KO) of a gene. The HDR route requires a homologous repair template, either endogenous, such as a sister chromatid, or exogenous, in the form of plasmid or single-stranded oligodeoxyribonucleotide (ssODN), and leads to precise repair or modification, such as a point mutation or gene fragment insertion.

As outlined above, the specificity of the system relies on the base pairing between the gRNA and the target sequence. The intrinsic problem of the system is, therefore, the off-target activity at *loci* exhibiting only partial complementarity to the gRNA. Indeed, it has been demonstrated that even several mismatches can be tolerated at some *loci*, generating considerable nonspecific mutational activity [8,9,10,11,12]. Moreover, even in the case of perfect complementarity, the cleavage efficiency varies significantly across the genome [13,14,15,16,17,18,19]. An additional problem is that the most widely used nuclease from *Streptococcus pyogenes* (SpCas9) also recognizes non-canonical PAMs such as NAG and NGA [10,12]. Many modifications of the system have been developed to circumvent these problems, including the use of rationally engineered Cas9 nucleases, paired nickases (Cas9n), orthologous nucleases, different delivery modalities and inducible systems, but no approach to date has shown a complete absence of the off-target effect [20,21,22,23,24,25,26,27,28]. Consequently, the essential task that the researcher must face in most of the CRISPR-Cas experiments is to design an experiment and to choose tools that lead to an acceptable ratio between on-target and off-target activity.

**Figure 1:**
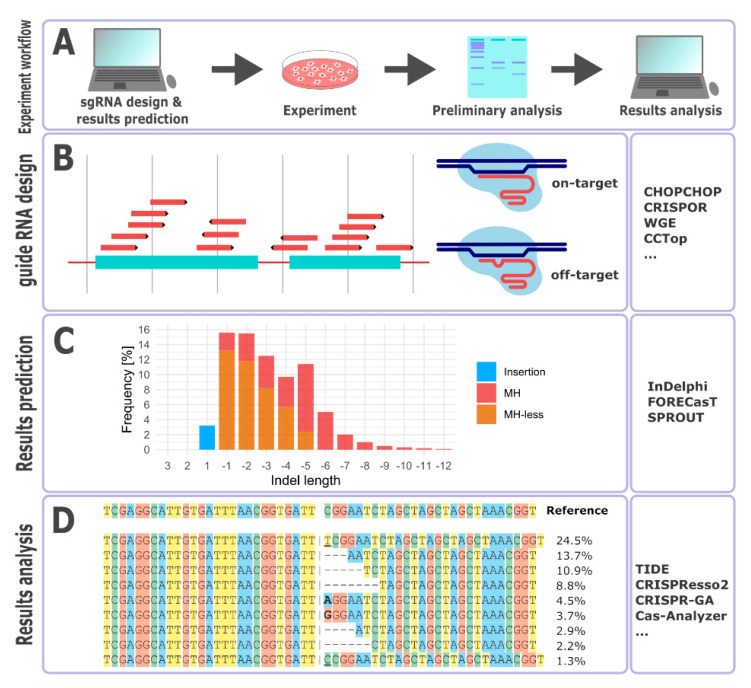
(**A**). The overview of a CRISPR-Cas experiment. Preliminary analysis involves screening of gRNA activity. Results analysis refers to NGS data analysis. (**B**). The gRNA design step involves the prediction of the gRNA efficiency (on-target activity) at a given *locus* and specificity (off-target activity); (**C**).The most probable results of CRISPR-Cas edits can be predicted by the analysis of microhomologies and by exploiting the known biases in the distribution of repair outcomes. (**D**). Web-based tools facilitating the post-experimental sequencing data analysis. MH—microhomology.

The rapid development in the field of genome engineering is reflected by the constantly expanding ecosystem of computational tools aimed at facilitating experimental design and result analysis. The main group of CRISPR-Cas computational tools is dedicated to aid in gRNA design by prediction of their efficiency (on-target activity) and specificity (off-target activity). These tools historically range from simple sequence alignment-based approaches to elaborate scorings of mismatches in any position of the gRNA and evaluation of the sequence features and are expanding progressively towards incorporation of the chromatin context and genetic variation data into the scoring algorithms. The relatively new group of tools exploits the observed biases in repair outcomes to predict the most likely results of a given CRISPR-Cas edit. The third class of tools is developed to assist in the evaluation of the editing results by analysis of the Sanger/next-generation sequencing (NGS) data. We present a schematic overview of CRISPR-Cas genome editing experiment as well as main groups of involved computational tools in Figure 1.

The large number of divergent tools inevitably leads to difficulty in finding the appropriate one for a specific type of experiment. There are several excellent reviews summarizing advancements in the field [29,30,31], but they are limited in their scope mainly to gRNA design. Furthermore, the rapid evolution and divergence in the area of CRISPR-Cas-related tools and in genome editing itself generates the need for constant updates.

Here, we present an updated overview of the currently available CRISPR-Cas-related tools from the perspective of a user who needs a convenient and reliable means to facilitate genome editing experiments at every step, from gRNA design to analysis of editing outcomes.

## 2. Experimental Design—the Choice of a Genome Editing System

Naturally, the research goals determine the model of choice, the targeted genomic site and the course of the experiment. There are, however, considerations particularly important in the context of CRISPR-Cas research. The crucial decisions concern the choice of Cas nuclease, format of CRISPR-Cas components and delivery of the system to the cell. The choices are particularly determined by the aim of the experiment (knockin, knockout, base editing, need for transient or stable nuclease activity), as well as the model used and its susceptibility to different forms of transfection or transduction.

SpCas9 is the most widely used nuclease in genome engineering, but advancements in the identification of its orthologs have resulted in a repertoire of nucleases characterized by different sizes, PAM requirements and cleavage patterns (e.g., Cas9s of *Staphylococcus aureus* and *Streptococcus thermophilus* or Cas12a of *Francisella novicida*) [32]. The other branch of research has resulted in “rationally engineered” nucleases with even more improved specificity [33]. The other strategies that proved useful in certain applications are the use of nickase (Cas9n) and catalytically inactive version of Cas9 (dCas9) [24,25,34,35]. Most of the gRNA design tools integrate PAM requirements and cleavage profile of many nucleases offering a considerable degree of flexibility in aiming at the desired editing site.

The format of CRISPR-Cas components (DNA, mRNA, RNP complex) may impact the efficiency and specificity of the nuclease. Purified Cas9 protein and in vitro transcribed gRNA can be combined to form the RNP complex delivered subsequently by electroporation or lipofection. This format leads to immediate yet short-lasting nuclease activity, as opposed to a more stable and longer expression from a plasmid. The peak of Cas9 activity delivered in the form of RNP complex occurs a few hours after transfection and then declines quickly as gRNA and Cas9 protein are degraded, leading to a significant increase in specificity [36,37]. A strategy based on a similar rationale utilizes in vitro transcribed Cas9 mRNA and gRNA [38,39].

Lipofection or calcium phosphate transfection are high-throughput and cost-effective delivery methods but may be not suitable in the case of difficult to transfect cells, such as primary or stem cells. Electroporation or viral transduction may be used instead but are more demanding in terms of labor and expense. Viral, specifically lentiviral, delivery allows a stable integration of genes into the genome. Adeno-associated viruses (AAVs) are preferable due to the relatively low immunogenicity but their packaging limit renders simultaneous delivery of sgRNA and SpCas9 gene problematic [40,41].

Another factor worth considering is that different cells exhibit a bias towards different DNA repair outcomes [3,6]. The phenomenon may be potentially levered to increase the chance of obtaining the desired type of edits.

Services such as Addgene or IDT offer not only plasmids, proteins and oligos necessary for CRISPR-Cas experiments but also repositories of protocols that the researcher may find useful at the experiment planning stage [42,43]. Springer Nature provides a browser of CRISPR-related methods papers, whereas Bio-protocol has released a curated collection of CRISPR-Cas-related protocols [44,45]. Both resources can be helpful in fulfilling more specific demands.

All the choices briefly outlined above need to be accounted for during the experiment planning and, specifically, guide RNA design. The tools described in the subsequent sections offer solutions appropriate for a given experimental approach. Nonetheless, the intrinsic uncertainty of in silico predictions, as well as variance in experimental conditions, make it necessary to empirically verify the efficiency and specificity of the Cas edits and to test more than one gRNA. The standard approach includes mismatch detection assays (T7E1 or SURVEYOR) or, preferentially, targeted NGS sequencing, but the repertoire of novel methods allowing evaluation of the editing efficiency is diverse [46,47,48,49,50,51]. The topic has been covered by extensive reviews of available methods [52,53,54].

## 3. Guide RNA Design

The problem of designing a specific and efficient gRNA may be avoided in certain cases by referring to existing libraries of validated gRNAs. These libraries offer assistance in choosing gRNAs with confirmed activity for specific applications; e.g., CRISPRlnc offers validated guides for lncRNAs, whereas CRISPRz is a database of validated CRISPR targets in zebrafish [55,56]. Additionally, Addgene delivers a list of prevalidated gRNA sequences for various species and applications, as well as genome-wide libraries of sgRNAs for gene knockout experiments [57,58].

However, these validated gRNAs are usually available only for frequently targeted *loci* such as GFP and for generic experiments. Additionally, to reproduce their activity, one needs to recreate the experimental condition, which is not necessarily possible or desirable. This limitation gives rise to a demand for custom gRNAs tailored for every particular purpose.

The main group of CRISPR-Cas computational tools is, therefore, dedicated to aid in gRNA design. At the moment of writing of this manuscript, the WeReview database indexes more than 60 tools in this category [59]. The programs usually score the possible guides according to the predicted cleavage efficiency (maximizing on-target activity) and specificity (minimizing off-target activity). The predictive models differ in the theory underlying the scoring methods and in generated results, forming a large and diverse category.

The observed variation in the activity of gRNAs has led to several attempts at identifying the relationship between the guide sequence characteristics and the efficiency of Cas cleavage. The problem was approached by conducting large-scale screening experiments analyzing the activity of hundreds or thousands of gRNAs at different *loci* by high-throughput methods [10,15,16,17,19,60,61,62,63]. These efforts resulted in sets of rules for the design of highly active gRNAs and predictive models that were subsequently implemented in public design tools (sgRNA Designer, SSC, CRISPRscan, sgRNA Scorer, CRISPRater) [16,17,19,60,62]. These models differ in weighting particular features, such as nucleotides preferred at every position, thermodynamics of secondary structures, occurrence of the same nucleotide tracts or GC content.

Doench et al. tested a pool of 1841 sgRNAs aiming at all possible targets of a panel of six mouse and three human genes coding cell surface markers and tested quantitatively their ability to generate biallelic knockouts by fluorescence-activated cell sorting (FACS), thereby separating the most active sgRNAs [63]. They identified sequence features that predict sgRNA efficacy: guanine was strongly preferred whereas cytosine was strongly unfavorable at position 20 counting from the 5′ end, the opposite was true for the position 16, and thymine was unfavored in the 3′ end region of the sgRNA which is related to the termination signal of the Pol III transcription machinery used to express the sgRNAs. Additionally, sgRNAs with low or high GC content appeared to be less active. The authors used the obtained data to determine sequence feature weights for activity predictions and built a predictive model for sgRNA activity by training a logistic regression classifier to identify sgRNAs exhibiting the highest activity for each gene using sequence features.

Doench et al. continued their research and proposed improvements to their model [16]. They used screening data generated with human and mouse genome-wide sgRNA libraries designed according to the previously proposed rules. They also included in the model additional features such as position-independent nucleotide counts, the location of the sgRNA target site within the gene, and thermodynamic properties of the sgRNAs. The authors designated the new model as Rule Set 2 and demonstrated its improved performance versus Rule Set 1 across different data sets. The Rule Set 2 was implemented subsequently in many gRNA design tools, such as CHOPCHOP, CRISPOR, GPP sgRNA Designer, or Benchling.

A different approach was proposed by Moreno-Mateos et al. [19]. They analyzed the molecular features that influence sgRNA activity and stability in vivo by using 1280 sgRNAs targeting 128 genes in the zebrafish genome. They injected in vitro transcribed sgRNAs together with Cas9-encoding mRNA into zebrafish embryos at the one-cell stage. The use of in vitro transcribed sgRNAs allowed the authors to focus on the influence of sgRNA stability and its relation to the sequence composition of the sgRNA. They demonstrated that more stable sgRNAs exhibit guanine enrichment and adenine depletion and, predictably, that the sgRNA stability positively correlates with the efficiency of the system. The authors identified also sequence features of the most active sgRNAs. The region spanning nucleotides 1–14 exhibited guanine enrichment, and positions 15–18 were characterized by cytosine enrichment. Thymidines and adenines were generally unflavoured, with the exception of positions 9 and 10. The authors applied randomized logistic regression to select the main features that determine the sgRNA efficiency. Then, they trained a linear regression model to predict the activity of a given sgRNA and implement it into the CRISPRscan tool [19].

It has been demonstrated, however, that the predictive accuracy of the many developed models is variable, and there is no superior algorithm in terms of reliability across different datasets [64,65]. The most plausible explanation for the observed inconsistency is that different experimental settings are used to generate the training datasets for every predictive model. A particular distinction identified to be of importance is the method of gRNA expression and delivery. As described earlier, the delivery modality (plasmid and viral vs. mRNA or RNP complex) can impact the cleavage kinetics and, consequently, the efficiency of edits. The majority of experiments were conducted in cell cultures and utilized the U6 promoter for relatively long-term and strong gRNA expression from a plasmid, whereas the other group of studies used the T7 promoter for in vitro transcription, and then, the gRNAs were injected into zebrafish embryos resulting in a short editing window. Thus, the general advice is to use a predictive algorithm that is based on an expression system analogous to the planned experiment. Specifically, Haeussler et al. suggested using the Doench score to rank the activity of gRNAs expressed from plasmids and the Moreno-Mateos score for gRNAs transcribed in vitro [64].

Another source of inconsistency among predictive algorithms is based on different approaches to gRNA activity assessment. A subset of studies used an indirect measurement and trained their models on data based on phenotypic changes, e.g., protein expression or drug resistance [16,63]. These models may be more appropriate in cases of functional studies but require reproduction of the original experimental conditions to achieve high accuracy. Another group of studies measured Cas9 activity directly by sequencing the targeted sites and by indel detection [19,60]. The applicability of the resulting models is therefore wider. Some tools integrate several models in their predictions (CRISPOR, CHOPCHOP, E-CRISP), allowing the user to evaluate different scores [64,66,67,68].

The problem of Cas9 off-target activity prediction was initially approached by using common sequence alignment algorithms such as BWA or Bowtie [68]. This approach offers, however, moderate usefulness due to the problem of falsely positive and falsely negative results. Algorithms miss targets with a high number of mismatches, leading to an omission of a proportion of potential off-target sites. On the other hand, they still deliver false positives, as many identified off-target sites are not actually cleaved. Tsai et al., demonstrated a considerable discrepancy between results predicted by the MIT CRISPR Design Tool (currently unavailable), E-CRISP, and the off-target sites empirically verified by GUIDE-seq [12]. Similar results were presented by Cameron et al., who compared predictions of Cas-OFFinder, CCTop and the off-target sites identified by Site-seq [69]. All these issues led, similarly to the problem of gRNA activity, to the development of more advanced scoring tools that aim to accurately identify the most likely off-target sites [10,16,61,70,71]. The algorithms were built on data from screening studies focused on the relationship between the observed Cas9 activity and the number and distribution of mismatches. The most widely applied algorithms are the MIT specificity score and the Cutting Frequency Determination (CFD) score [10,16].

Since all these tools were developed on the basis of different data-sets and differ in the way that they score particular factors, it is advisable to compare results generated by different algorithms. Certain tools implement more than one predictive algorithm, allowing the user to choose the most appropriate approach (CRISPRseek, CRISPRscan, CRISPR Multi-Targeter, CRISPOR).

The majority of available tools still use only the basic sequence features to predict off-target activity of a given gRNA. The involvement of more complex characteristics, such as genomic context, chromatin environment, DNA/RNA thermodynamics, and the occurrence of DNA or RNA bulges, is ambiguous and not fully understood. There are, however, attempts to incorporate these factors into predictive models, e.g., CROP-IT, DeepCRISPR, uCRISPR or CRISTA [72,73,74,75].

Among tools facilitating the design of efficacious and specific sgRNAs, we recommend CRISPOR. It integrates the Doench score, in our opinion one of the most robust efficiency prediction systems, along with many other algorithms, allowing a direct comparison of their results, making the prediction more reliable. It supports 15 nucleases and more than 150 genomes. CRISPOR provides sequences for in vitro expression or cloning of a given sgRNA using different systems as well as primers for amplification of on- and off-target sites. The tool integrates also the Bae et al., 2014 predictions of repair profile and the Chen et al., 2018 frameshift prediction [5,76]. Moreover, CRISPOR warns the user when a designed gRNA contains motifs that should be avoided as described by Graf et al. [77].

In Supplementary Table S1, we present a summary of the most popular tools for gRNA design.

## 4. Repair Outcome Predictions

The relatively new branch of tools utilizes the fact that DNA repair is, to a certain extent, a deterministic process. The tools allow us to predict Cas edit results by the analysis of microhomologies and by exploiting the biases in the repair outcome. High-fidelity prediction of DSB repair outcomes is one of crucial concerns in CRISPR-Cas experimental design. Depending on the purpose of genome editing different repair products are desired, such as frame shift indels used for gene knockout or, contrarily, in-frame deletions maintaining the transcriptional activity of the gene. Genome editing outcomes are determined by the DNA repair pathway, which is in turn influenced by many factors, including the cell cycle stage and repair-related proteins expressed in a given cell type [78]. The most common DSB repair mechanisms, canonical nonhomologous end joining (c-NHEJ) and alternative nonhomologous end joining (alt-NHEJ), including microhomology mediated end joining (MMEJ), often lead to insertion or deletion at the cutting site [79]. It has been proven that the distribution of repair outcomes is not random but strongly reproducible within a cell population and depends mainly on the targeted sequence. Two dominant categories of mutations occurring in the effect of DSB repair has been revealed: NHEJ-dependent single base insertion (duplication of nucleotide in position -4 from PAM) and MH-mediated deletion. [4,6,80,81] These observations were adopted by the developers of bioinformatic tools dedicated to detailed characterization of repair outcomes and improve experimental design. A comparison of these tools is presented in Table 1 [3,7,82].

Allen et al. and Shen et al. created online tools for Cas9 edit result prediction (FORECasT and InDelphi, respectively) based on similar experiments [3,7]. Both groups used artificial constructs containing a gRNA expressed under the human U6 promoter and target sequence complementary to the guide flanked by different nucleotide contexts. Dozens of these constructs were transfected into cell lines stably expressing Cas9, leading to edits of the provided target sites [3,7]. Leenay et al., used an endogenous model to develop an online tool named SPROUT. The authors targeted 1656 genomic *loci* in primary CD4^+^ T cells isolated from 18 human donors and used an RNP complex containing crRNA, tracrRNA and the Cas9 protein [82]. In each experiment, cut-site-proximal repair products were characterized after PCR amplification of target sites with their flanking regions and deep-sequencing, revealing a sequence-dependent mutation profile. Then, data from sequencing were used to train algorithms developed by the authors to precisely predict DSB repair outcomes, and subsequently, the accuracy of prediction was tested. Each algorithm exhibited high accuracy of repair profile prediction for a given cell type [82].

The algorithms developed by authors are available online. SPROUT requires only sequences of the gRNA and PAM (total of 23 nt) to output information regarding predicted results, including the fraction of total reads with insertion, insertion to deletion ratio, average insertion and deletion length and the inserted base pair. SPROUT does not show any information about sequences of outcomes [82]. FORECasT requests the target DNA sequence and a location of PAM to output sequences of the ten most likely results and percentage of in-frame mutations [3]. InDelphi provides many capabilities and information not presented in other tools. Aside from sequences of predicted outcomes with their frequency, this tool presents information on the frameshift frequency and microhomology (MH) strength of a target site. Predicted deletions are described as microhomology-resulted or microhomology-less. Moreover, InDelphi allows choosing the cell type of interest, HEK293, HCT116, K562, mESC and U2OS, which is required to achieve a high accuracy of prediction. Furthermore, in batch mode, InDelphi allows quick searching for up to 80 gRNAs followed by a chosen PAM in the input sequence and comparing their precision, frameshift frequency and microhomology strength with the results of a single mode for each gRNA. In the gene mode InDelphi scans exonic sequences of selected human or mouse genes to provide hundreds of gRNAs with statistics similar to those in batch mode, and includes information about distances from the 5′ and 3′ ends of the targeted exon for each gRNA [7].

**Figure 2:**
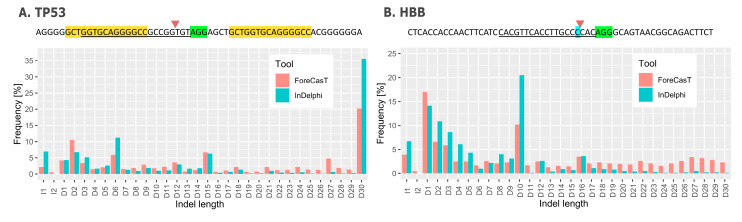
Editing outcome predictions generated by ForeCasT and InDelphi with a relevant sequence presented above. (**A**). Results obtained with gRNA targeting the human tumor protein P53 (*TP53*) gene; (**B**). Results obtained with gRNA targeting the human hemoglobin β (*HBB*) gene. Green rectangles—PAM sequences, yellow rectangles—tracts of microhomology, blue rectangle—duplicated nucleotide, red triangles—cutting sites.

We compared predictions generated by ForeCast and InDelphi to empirical data obtained in genome editing experiments carried out at two distinct loci. The sites differ in nucleotide sequence architecture: the *TP53* (tumor protein P53) locus is microhomology-rich and contains homologous tracts in close proximity to the cleavage site (Figure 2A, marked in yellow) making MMEJ a highly likely mechanism of DNA repair. In the targeted region of *HBB* (hemoglobin β) longer microhomologies are relatively less abundant.

In the case of *TP53*, both algorithms correctly predicted the occurrence of a relatively long, 30 bp deletion as the most probable editing result. Indeed, we observed a biallelic deletion, 30 and 50 bp long at this site in HEK293T cells [47]. The 50 bp deletion was not predicted since it exceeds the indel length capacity of both tools. The results confirm that MMEJ-based repair may be harnessed for highly predictable and precise genome editing. In the case of *HBB*, neither FOREcasT nor InDelphi was able to accurately assess the probability distribution across the range of indels. As reported by Cradick et al. introduction of a DSB resulted in an exceptionally high frequency of a one nucleotide insertion at the locus (43.2%) in HEK293T cells, whereas the tools assigned its relatively low probability (3.9% vs. 6.7%, ForeCasT and InDelphi, respectively) [83]. Nonetheless, both tools were able to predict which nucleotide is the most probable to be inserted (cytidine). The principles determining the preference of a nucleotide to be inserted appear to be relatively straightforward—the inserted base is usually homologous to the nucleotide at position −4 from PAM (Figure 2B, marked in blue) [3,4]. The high frequency of single nucleotide deletions predicted by both algorithms stems from the occurrence of CCCC motif directly at the cutting site. It has been reported that cytosine at the −5 and −4 positions relative to the cutting site results in a high probability of a single nucleotide excision [3,4]. However, the real frequency of this event turned out to be significantly lower (6.8%) [83].

The examples presented above point to the conclusion that, in spite of the considerable progress achieved in the area emerging from the intersection of CRISPR-Cas technology and DNA repair research, there is still room for improvement in terms of the ability to reliably predict DNA editing outcomes. Longer microhomologies are a strong predictive factor of MMEJ engagement and the occurrence of predictable deletions. In cases when the editing precision is essential it may be reasonable to include the presence of microhomologies as a criterion for choosing a site to be edited. It narrows the targetable sequence range significantly, but the achieved accuracy may compensate for this restriction. The uncertainty intrinsic for in silico predictions notwithstanding, the idea of accurate prediction of DNA edits may prove exceptionally useful in the field of genome engineering.

## 5. Outcomes Analysis

Another group of algorithms is designed to facilitate the postexperiment (posterior) analysis of the CRISPR-Cas9 edition outcomes, including off-target assessment. Essentially, the postexperiment analysis algorithms enable quantification and visualization of CRISPR-Cas9 outcomes in tabular view with relevant graphs. These algorithms show the frequency of indels and repair types (NHEJ or HDR) simplifying data interpretation.

Some of tool such as Tracking of Indels by Decomposition (TIDE) are intended to analyze Sanger sequencing results of the targeted *locus*, providing a comprehensive profile of all insertions and deletions that occurred in a sample [49]. The redesigned version of this tool (TIDER—Tracking of Insertions, Deletions and Recombination events) also enables estimations of the efficacy of templated genome editing and assesses the frequency of HDR events [84]. The advantage of these approaches is that they require basic and easily accessible laboratory equipment and are relatively inexpensive. The other group of programs is concerned with NGS data analysis. Tools such as CRISPRpic, CRIS.py or CrispRVariants provide a command-line interface, which enables a detailed analysis of the results but may be difficult to approach for less experienced users [85,86,87,88]. Therefore, to address this issue, various web-based, command line-less and user-friendly programs for NGS analysis have been developed.

CRISPR-Genome Analyzer (CRISPR-GA) uses NGS data to quantify and characterize indels and homologous recombination events at the targeted sites [89]. Its pipeline consists of quality control and alignment of reads, which is subsequently used to compute the editing efficiency and frequency of HDR and NHEJ and to create charts with indel localizations and sizes.

Another tool available online is Cas-Analyzer, a JavaScript-based implementation for NGS data analysis [88]. This program provides results similar to those of CRISPR-GA but has one practical advantage: avoiding of a time-consuming step of data upload to a server. The Cas-Analyzer algorithm runs wholly on the side of a web browser, leading to a significant time savings. Moreover, it supports various nucleases used by scientists, including the paired nickases approach.

One additional program that facilitates the analysis of sequencing data is CRISPResso2, an updated version of the CRISPResso tool [90,91]. CRISPResso2, aside from performing edit outcome frequency assessment, enables allele-specific quantification of results by aligning reads to multiple reference sequences and computing statistics separately for each allele. Furthermore, CRISPResso2 may be very useful for base editing assays due to its detailed analysis of substitutions. The main disadvantage of this tool is the limit of data that can be uploaded for an analysis (up to 100 Mb in one FASTQ file). However, the command line version of CRISPResso2 containing additional tools and no data upload limits is available online. We compared the main features of the described tools in Table 2.

There are also tools dedicated to the analysis of NGS results from pooled screening experiments. Several tools developed for such purposes such as like MAGeCK and BAGEL utilize a command line interface, which may limit their accessibility for less experienced users [93,94]. Newly developed programs that offer web-based and user-friendly interfaces for the deconvolution of pooled screening data, such as CRISPRAnalyzeR, CRISPRcloud2 and PinAPL-Py, are addressed for researchers with zero programming background [95,96,97]. Each of these platforms requires uploading FASTQ files with NGS data and a gRNA library file (predefined gRNA libraries are also provided on websites) to start the workflow. The output of the mentioned tools includes information regarding quality control, detailed statistical analysis and visualization of the data. Moreover, CRISPRAnalyzeR and CRISPRcloud2 offer tutorial videos on their websites, simplifying the usage of these tools.

The methods described above can be used to analyze editing outcomes at on-target sites as well as at the off-target sites predicted in silico. This approach is, however, biased towards the predicted sites, and, usually, only the most probable cleavage sites are actually assayed. This, consequently, poses the risk of omitting the unpredicted or low-likely cleavages. The solution for this issue is to apply whole-genome sequencing (WGS) but the technique is expensive and unsuitable for analysis of many clones, additionally, it requires high coverage to detect low-frequency edits. All these problems resulted in the development of a distinct group of methods dedicated to cleavage detection across the entire genome without the requirement for WGS data. This group consists of techniques used to identify DSBs generated in living cells and in cell-free in vitro systems. The first type of methods includes unbiased identification of double-strand breaks evaluated by sequencing (GUIDE-seq), High-Throughput, Genome-wide translocation Sequencing (HTGTS), Integration-Deficient Lentiviral Vector (IDLV), direct in situ Breaks Labeling, Enrichment on Streptavidin, and next-generation Sequencing (BLESS) and Discovery of In Situ Cas Off-targets and VERification by sequencing (DISCOVER-seq) [12,98,99,100,101]. The second type consists of Selective enrichment and Identification of Tagged genomic DNA Ends by sequencing (SITE-Seq) and Circularization for In vitro Reporting of CLeavage Effects by sequencing (CIRCLE-seq) [69,102]. The latter group has the advantage of eliminating the necessity for cell culture and transfection of cells with components of the CRISPR-Cas system. In spite of different technical approaches, all these methods rely eventually on NGS sequencing. Some of these techniques are supplemented with dedicated computational pipelines facilitating the data analysis such as BLENDER (BLunt End finder) used for identification of off-target edits sites by ChIP-Seq in DISCOVER-seq method [101].

## 6. Discussion

Computational tools serve as valuable support for genome editing experiments. The significant progress in the development of scoring algorithms notwithstanding, the majority of predictive models relay on relatively basic sequence features. There is still room for improvement in terms of the understanding of the exact role of chromatin structure, nucleic acid thermodynamics or the occurrence of bulges in the process of Cas9 cleavage. As mentioned earlier, the issue of chromatin state and DNA accessibility is considered one of the key factors to be analyzed in the context of either the efficiency or specificity of CRISPR-Cas9 cleavage. Indeed, the association between Cas9 cleavage efficiency and chromatin accessibility has been demonstrated [103,104,105,106,107,108,109]. Avoidance of, e.g., strongly positioned nucleosomes may be beneficial in terms of efficacious edits and reliable assessment of the off-target risk.

Another important problem is naturally occurring genetic variation. The majority of gRNA design tools refer to the human reference genome and do not include variant information. Thus, SNPs and mutations in target sequences may have considerable effects on cleavage specificity either by disrupting the targeted sequence or by creating unexpected off-target sites [110]. These effects may, in turn, lead to unintended edits of essential genes, e.g., tumor suppressors. In fact, it has been demonstrated that genetic variation leads to a wide distribution of sites containing unexpected PAMs and absent PAMs across the genome [102,111,112]. Certain tools incorporate variation information into the process of gRNA design (CRISPOR, WGE, E-CRISP, CCTop). Moreover, there are tools dedicated to variant-specific CRISPR-Cas9 edits (SNP-CRISPR, AlleleAnalyzer) [113,114].

Generally, we suggest using tools that offer more than one scoring algorithm to assess the activity of gRNAs as accurately as possible. By this criterion CRISPOR, is the most advisable tool. Additionally, as we described earlier, the variety of additional features makes this tool particularly useful. Nevertheless, the activity and specificity of any custom gRNA need to be empirically validated due to the reported inconsistency of the models, as well as the risk of unexpected genetic variation or heterogeneity of cell populations. Another point to bear in mind is the fact that some models are not universally applicable. For example, the Moreno-Mateos score is best suited for experiments with in vitro expressed gRNAs.

At the moment of the manuscript writing, InDelphi is the most advanced predictive tool, providing the deepest analysis of results and several utilities that may be helpful in outcomes analysis. However, the data that the predictive algorithms are built on are limited to only certain cell lines, offering much less reliable estimation for cells with different genetic backgrounds. Cell line-specific DNA repair machinery disruption is a phenomenon that may lead to differential patterns of results obtained among different cell lines. Another issue worth consideration is the fact that accuracy of the predictive tools is moderate in the case of “difficult” sequences, such as long tracts of repeated sequences.

Of the tools aimed at post experiment results analysis, TIDE is the most straightforward and approachable solution; however, it has important limitations. Its accuracy is reduced in the case of low-frequency outcomes, and because it is a PCR-based method, it may not be well suited for the analysis of sequences containing tracts of tandem repeats. Additionally, the maximum size of indels that this tool detects is limited to 50 base pairs, making it not applicable in the analysis of larger rearrangements.

The outlined limitations may be overcome by applying tools based on NGS data analysis such as CRISPR-GA or CRISPResso. The downsides of these approaches are, however, tied to the inherent limitations of NGS techniques. NGS is a costly and time-consuming system and, in many cases, requires the use of an external service. Despite these issues, NGS is still a method of choice for a reliable analysis of CRISPR-Cas experimental results.

For a quick search of tools useful in the field of CRISPR-Cas experiments we recommend the WeReview: CRISPR Tools website [59]. It is a large, user-updated repository of computational tools related to CRISPR-Cas research. It offers an extensive list of services categorized by main characteristics, searching and sorting options, yet the presented features may be too basic for advanced users.

## Figures and Tables

**Table 1:** Comparison of repair outcome prediction tools.

Compared Feature	FORECasT	SPROUT	inDelphi
Indel frequency	YES	YES	YES
Average indel length	NO	YES	NO
Sequences of predictions	YES	NO	YES
Alignment of predicted sequences	YES	NO	YES
Frequency count for every predicted sequence	YES	NO	YES
Frameshift/ in frame frequency	YES	NO	YES
MH strength of target site	NO	NO	YES
Level of homogeneity of predictions	NO	NO	YES
Distinction between MH and MH-less deletions	NO	NO	YES
Cell type enable to choose	NO	NO	YES (HEK293, HCT116, K562, mESC, U2OS)
Batch mode	Available in command line tool	NO	YES
Gene mode	NO	NO	YES (for human and mouse)
Shareable link to results	NO	NO	YES
Summary statistics for download	YES	NO	YES
References	[3]	[82]	[7]

**Table 2:** Comparison of web-based postexperimental analysis tools.

Compared Feature	CRISPResso2	Cas-Analyzer	CRISPR-GA	TIDE/TIDER
Type of analysis	NGS	NGS	NGS	Sanger sequencing
File type	FASTQ	FASTQ	FASTQ	ABI
Output	-indel sizes and positions -HDR/NHEJ frequency -sequence alignment with reference -allele specific quantification	-indel sizes and positions -HDR/NHEJ frequency -sequence alignment with reference	-indel sizes and positions -HDR/NHEJ frequency	-indel sizes and positions -HDR/NHEJ frequency
Batch functionality	YES	NO	NO	NO
Base editing experiments	YES	NO	NO	NO
Template-mediated editing	YES	YES	YES	YES (TIDER)
Supported nucleases	Cas9, Cpf1	SpCas9, StCas9, NmCas9, SaCas9, CjCas9, AsCpf1/LbCpf1, paired nucleases: ZFNs, TALENs, Cas9 nickases, dCas9-FokI	Cas9	SpCas9, SaCas9, St1Cas9, NmCas9, AsCpf1, FnCpf1, LbCpf1
Need to upload dataset to a server	YES (up to 100Mb)	NO	YES	YES
Command line interface tool available	YES	NO	NO	NO
References	[90]	[88]	[89]	[49,92]

## Supplementary Materials

The following are available online at https://www.mdpi.com/2073-4409/9/5/1288/s1, Table S1: The most popular guide RNA design tools and their main features.
