# Supplementary material for: Computational Tools and Resources Supporting CRISPR-Cas Experiments
Source: Cells. 2020 May 22;9(5):1288. doi: 10.3390/cells9051288 (PMC7290941; doi:10.3390/cells9051288)
Supplement: Supplementary file 1 [file cells-09-01288-s001.pdf]

Supplementary Table 1. The most popular guide RNA design tools and their main features.

Tool	Input	Enzyme	Organism	On-Target Scoring Method	Off-Target Scoring Method	Features	Referenc e
CHOPCHOP	GeneID Coordinates Sequence	SpCas9; SpCas9n; Cas12a (Cpf1); CasX; Cas13 (C2C2); TALEN	Variety	Doench et al. 2014; Doench et al. 2016; Chari et al. 2015; Xu et al. 2015; Moreno-Mateos et al. 2015; G20	MIT specificity score; Cong et al., 2013	Designs primers for the edited site amplification; restriction sites map; exon-intron map; Integrates Shen et al. 2018 predictions of repair profile	[1,2]
CRISPOR	Coordinates Sequence	SpCas9; SpCas9-HF1; eSpCas9 1.1; ScCas9; iSpyMacCas9; SaCas9; xCas9; SaCas9-KKH; SpCas9-VQR; NmeCas9; SpCas9-VRER; StCas9; CjCas9; AsCas12a (Cpf1); LbCas12a (Cpf1)	Variety	Doench et al. 2016 Chari et al. 2015; Xu et al. 2015; Wu-Crisp Doench et al. 2014; Wang et al. 2014 Moreno-Mateos et al. 2015; Azimuth in-vitro crisprRank	MIT Specificity Score; CFD Specificity score	Designs primers for the edited site amplification; restriction sites map; provides sequences for in vitro expression or cloning of designed sgRNAs; Integrates Bae et al. 2014 predictions of repair profile and Chen et al. 2018 frameshift prediction	[3]
CRISPRscan	GeneID Sequence	SpCas9; AsCas12a (Cpf1); LbCas12a (Cpf1)	Variety	Moreno-Mateos et al. 2015;	MIT Specificity Score; CFD Specificity score; Cong et al., 2013	Appropriate for in-vitro guide RNA transcription under T7/Sp6 promoter; includes genetic variation;	[4]
IDT	GeneID Sequence	SpCas9	*Homo sapiens* *Mus musculus* *Rattus norvegicus* *Danio rerio* *Caenorhabditis elegans*	Own	Own	Offers predesigned guide RNAs, design of custom guide RNAs and checking of designed guide RNAs	[5]
WGE	GeneID Sequence	SpCas9	*Homo sapiens* *Mus musculus*	Hodgkins et al. 2015	Hodgkins et al. 2015	Integrates Genoverse genome browser; exon-intron map; protein map; includes genetic variation; integrates Allen et al. 2019 predictions of repair profile	[6]
Benchling	GeneID Coordinates Sequence	SpCas9; NmeCas9; StCas9; TdCas9; SaCas9; AsCas12a (Cpf1); LbCas12a (Cpf1);	Variety	Doench et al. 2014; Doench et al. 2016;	MIT Specificity Score	Allows design of guides for base editing; restriction sites map; exon-intron map; protein map	[7]
GPP sgRNA Designer (CRISPick)	GeneID Sequence	SpCas9; SaCas9; AsCas12a (Cpf1); enCas12a (Cpf1)	*Homo sapiens* *Mus musculus* *Rattus norvegicus*	Doench et al. 2016;	CFD Specificity score	Includes genetic variation	[8–10]
CROP-IT	Coordinate Sequence	SpCas9	*Homo sapiens* *Mus musculus*	Singh et al. 2015	Singh et al. 2015	Incorporates chromatin state information	[11]
CRISTA	Coordinate Sequence	SpCas9	Variety	Abadi et al. 2017	BWA; Abadi et al. 2017	Incorporates advanced parameters in the prediction: genomic context, RNA	[12]

						thermodynamics, occurrence of bulges	
E-CRISP	GeneID Sequence	SpCas9	Variety	Heighwer et al. 2014; Doench et al. 2014; Xu et al. 2015	Bowtie2	Includes genetic variation	[13]
sgRNA Scorer 2.0	Sequence	SpCas9; SaCas9; AsCas12a (Cpf1); NmeCas9; StCas9	Variety	Chari et al., 2015; Chari et al., 2017	Aach et al. 2014		[14,15]
CasFinder	Coordinate Sequence	SpCas9; StCas9; NmeCas9	*Homo sapiens* *Mus musculus*	-	Aach et al. 2014	Exome-wide catalog of Cas9 cleavage sites	[16]
CCTop	Sequence	SpCas9; SpCas9-VQR; SpCas9-VRER; AsCas12a (Cpf1); LbCas12a (Cpf1); FnCas12a (Cpf1); SaCas9; StCas9; NmeCas9;TdCas9	Variety	CRISPRater	Stemmer et al. 2017	Includes genetic variation	[17,18]
DeepCRISPR	Sequence	SpCas9	Homo sapiens	Chuai et al. 2018	Chuai et al. 2018	Integrates the epigenetic information in different cell types	[19]
SNP-CRISPR	csv file containing genomic coordinates and variants	SpCas9	*Homo sapiens* *Mus musculus* *Danio rerio* *Drosophila melanogaster* *Rattus norvegicus*	Housden et al. 2015	BLAST	Dedicated to variant-specific CRISPR-Cas9 edits	[20,21]
AlleleAnalyzer	BCF/VCF file with genotypes	SpCas9; SaCas9	*Homo sapiens*	Keough et al. 2019	CRISPOR	An open-source Python software tool; dedicated to variant-specific CRISPR-Cas9 edits	[22]

AsCas12a—*Acidaminococcus spp.* Cas2a; CjCas9—*Campylobacter jejuni* Cas9; eSpCas9 1.1—enhanced specificity *Streptococcus pyogenes* Cas9 1.1; iSpyMacCas9—increased *Streptococcus pyogenes* and *Streptococcus macacae* Cas9; LbCas12a—*Lachnospiraceae spp.* Cas12a; NmeCas9—*Neisseria meningitidis* Cas9; SaCas9—*Staphylococcus aureus* Cas9; SaCas9-KKH—*Staphylococcus aureus* Cas9 KKH mutant; ScCas9—*Streptococcus canis* Cas9; SpCas9—*Streptococcus pyogenes* Cas9; SpCas9-HF1 – *Streptococcus pyogenes* Cas9 High Fidelity 1; SpCas9n—*Streptococcus pyogenes* Cas9 nickase mutant; SpCas9-VQR—*Streptococcus pyogenes* Cas9 VQR mutant; SpCas9-VRER—*Streptococcus pyogenes* Cas9 VRER mutant; StCas9—*Streptococcus thermophilus* Cas9; TALEN—Transcription Activator-Like Effector Nuclease; TdCas9—*Treponema denticola* Cas9; xCas9—expanded PAM SpCas9.

References

1. Montague, T.G.; Cruz, J.M.; Gagnon, J.A.; Church, G.M.; Valen, E. CHOPCHOP: a CRISPR/Cas9 and TALEN web tool for genome editing. *Nucleic Acids Res* **2014**, *42*, W401–W407, doi:10.1093/nar/gku410.
2. Labun, K.; Montague, T.G.; Gagnon, J.A.; Thyme, S.B.; Valen, E. CHOPCHOP v2: a web tool for the next generation of CRISPR genome engineering. *Nucleic Acids Res* **2016**, *44*, W272–W276, doi:10.1093/nar/gkw398.
3. Haeussler, M.; Schönig, K.; Eckert, H.; Eschstruth, A.; Mianné, J.; Renaud, J.-B.; Schneider-Maunoury, S.; Shkumatava, A.; Teboul, L.; Kent, J.; et al. Evaluation of off-target and on-target scoring algorithms and integration into the guide RNA selection tool CRISPOR. *Genome Biology* **2016**, *17*, 148, doi:10.1186/s13059-016-1012-2.
4. Moreno-Mateos, M.A.; Vejnar, C.E.; Beaudoin, J.-D.; Fernandez, J.P.; Mis, E.K.; Khokha, M.K.; Giraldez, A.J. CRISPRscan: designing highly efficient sgRNAs for CRISPR-Cas9 targeting in vivo. *Nat Methods* **2015**, *12*, 982–988, doi:10.1038/nmeth.3543.

5. Custom Alt-R® CRISPR-Cas9 guide RNA | IDT Available online: https://eu.idtdna.com/site/order/designtool/index/CRISPR_CUSTOM (accessed on Feb 13, 2020).
6. Hodgkins, A.; Farne, A.; Perera, S.; Grego, T.; Parry-Smith, D.J.; Skarnes, W.C.; Iyer, V. WGE: a CRISPR database for genome engineering. *Bioinformatics* **2015**, *31*, 3078–3080, doi:10.1093/bioinformatics/btv308.
7. Cloud-Based Informatics Platform for Life Sciences R&D Available online: https://www.benchling.com/ (accessed on Feb 13, 2020).
8. Doench, J.G.; Fusi, N.; Sullender, M.; Hegde, M.; Vaimberg, E.W.; Donovan, K.F.; Smith, I.; Tothova, Z.; Wilen, C.; Orchard, R.; et al. Optimized sgRNA design to maximize activity and minimize off-target effects of CRISPR-Cas9. *Nat Biotechnol* **2016**, *34*, 184–191, doi:10.1038/nbt.3437.
9. Kim, H.K.; Min, S.; Song, M.; Jung, S.; Choi, J.W.; Kim, Y.; Lee, S.; Yoon, S.; Kim, H. (Henry) Deep learning improves prediction of CRISPR–Cpf1 guide RNA activity. *Nat Biotechnol* **2018**, *36*, 239–241, doi:10.1038/nbt.4061.
10. Sanson, K.R.; DeWeirdt, P.C.; Sangree, A.K.; Hanna, R.E.; Hegde, M.; Teng, T.; Borys, S.M.; Strand, C.; Joung, J.K.; Kleinstiver, B.P.; et al. Optimization of AsCas12a for combinatorial genetic screens in human cells. *bioRxiv* **2019**, 747170, doi:10.1101/747170.
11. Singh, R.; Kuscu, C.; Quinlan, A.; Qi, Y.; Adli, M. Cas9-chromatin binding information enables more accurate CRISPR off-target prediction. *Nucleic Acids Res* **2015**, *43*, e118–e118, doi:10.1093/nar/gkv575.
12. Abadi, S.; Yan, W.X.; Amar, D.; Mayrose, I. A machine learning approach for predicting CRISPR-Cas9 cleavage efficiencies and patterns underlying its mechanism of action. *PLOS Computational Biology* **2017**, *13*, e1005807, doi:10.1371/journal.pcbi.1005807.
13. Heigwer, F.; Kerr, G.; Boutros, M. E-CRISP: fast CRISPR target site identification. *Nat Methods* **2014**, *11*, 122–123, doi:10.1038/nmeth.2812.
14. Chari, R.; Mali, P.; Moosburner, M.; Church, G.M. Unraveling CRISPR-Cas9 genome engineering parameters via a library-on-library approach. *Nat Methods* **2015**, *12*, 823–826, doi:10.1038/nmeth.3473.
15. Chari, R.; Yeo, N.C.; Chavez, A.; Church, G.M. sgRNA Scorer 2.0: A Species-Independent Model To Predict CRISPR/Cas9 Activity. *ACS Synth. Biol.* **2017**, *6*, 902–904, doi:10.1021/acssynbio.6b00343.
16. Aach, J.; Mali, P.; Church, G.M. CasFinder: Flexible algorithm for identifying specific Cas9 targets in genomes. *bioRxiv* **2014**, 005074, doi:10.1101/005074.
17. Labuhn, M.; Adams, F.F.; Ng, M.; Knoess, S.; Schambach, A.; Charpentier, E.M.; Schwarzer, A.; Mateo, J.L.; Klusmann, J.-H.; Heckl, D. Refined sgRNA efficacy prediction improves large- and small-scale CRISPR–Cas9 applications. *Nucleic Acids Res* **2018**, *46*, 1375–1385, doi:10.1093/nar/gkx1268.
18. Stemmer, M.; Thumberger, T.; Keyer, M. del S.; Wittbrodt, J.; Mateo, J.L. CCTop: An Intuitive, Flexible and Reliable CRISPR/Cas9 Target Prediction Tool. *PLOS ONE* **2015**, *10*, e0124633, doi:10.1371/journal.pone.0124633.
19. Chuai, G.; Ma, H.; Yan, J.; Chen, M.; Hong, N.; Xue, D.; Zhou, C.; Zhu, C.; Chen, K.; Duan, B.; et al. DeepCRISPR: optimized CRISPR guide RNA design by deep learning. *Genome Biology* **2018**, *19*, 80, doi:10.1186/s13059-018-1459-4.
20. Chen, C.-L.; Rodiger, J.; Chung, V.; Viswanatha, R.; Mohr, S.E.; Hu, Y.; Perrimon, N. SNP-CRISPR: A Web Tool for SNP-Specific Genome Editing. *G3: Genes, Genomes, Genetics* **2020**, *10*, 489–494, doi:10.1534/g3.119.400904.
21. Housden, B.E.; Valvezan, A.J.; Kelley, C.; Sopko, R.; Hu, Y.; Roesel, C.; Lin, S.; Buckner, M.; Tao, R.; Yilmazel, B.; et al. Identification of potential drug targets for tuberous sclerosis complex by synthetic screens combining CRISPR-based knockouts with RNAi. *Sci. Signal.* **2015**, *8*, rs9–rs9, doi:10.1126/scisignal.aab3729.
22. Keough, K.C.; Lyalina, S.; Olvera, M.P.; Whalen, S.; Conklin, B.R.; Pollard, K.S. AlleleAnalyzer: a tool for personalized and allele-specific sgRNA design. *Genome Biology* **2019**, *20*, 167, doi:10.1186/s13059-019-1783-3.
